# Evolution and ecology of *Jeilongvirus* among wild rodents and shrews in Singapore

**DOI:** 10.1186/s42522-023-00094-1

**Published:** 2023-12-18

**Authors:** Lena Ch’ng, Dolyce H.W. Low, Sophie A. Borthwick, Rong Zhang, Zoe A. Ong, Yvonne C.F. Su, Alan T. Hitch, Gavin J.D. Smith, Ian H. Mendenhall

**Affiliations:** 1 Programme in Emerging Infectious Diseases, Duke-NUS Medical School, Singapore, 169857 Singapore; 2 Museum of Wildlife and Fish Biology, Department of Wildlife, Fish and Conservation Biology, University of California at Davis, Davis, CA 95616 USA; 3 Centre for Outbreak Preparedness, Duke-NUS Medical School, Singapore, 169857 Singapore

**Keywords:** Metagenomics, Phylogeny, Rodent paramyxovirus, Urban disease ecology

## Abstract

**Background:**

*Jeilongvirus* was proposed as a new genus within the *Paramyxoviridae* in 2018. The advancement in metagenomic approaches has encouraged multiple reports of *Jeilongvirus* detection following the initial species discovery, enriching species diversity and host range within the genus. However, *Jeilongvirus* remains understudied in Singapore, where interfaces between humans and small mammals are plentiful.

**Methods:**

Here, we utilized metagenomic sequencing for the exploration of viral diversity in small mammal tissues. Upon discovery of *Jeilongvirus*, molecular screening and full genome sequencing was conducted, with the data used to conduct statistical modelling and phylogenetic analysis.

**Results:**

We report the presence of *Jeilongvirus* in four species of Singapore wild small mammals, detected in their spleen and kidney. We show that full genomes of three Singapore *Jeilongvirus* encode for eight ORFs including the small hydrophobic and transmembrane proteins. All generated genomes cluster phylogenetically within the small mammal subclade, but share low genetic similarity with representative *Jeilongvirus* species. Statistical modelling showed no spatial or temporal patterns and differences among species, life history traits and habitat types.

**Conclusions:**

This study serves as a basis for understanding dynamics between *Jeilongvirus* and small mammal hosts in Singapore by displaying the virus generalist nature. In addition, the initial detection can help to invoke improved routine surveillance and detection of circulating pathogens in synanthropic hosts.

**Supplementary Information:**

The online version contains supplementary material available at 10.1186/s42522-023-00094-1.

## Background

The family *Paramyxoviridae* consists of enveloped, negative-sense RNA viruses with genomes ranging from 14 kb to 20 kb in length. The non-segmented genomes encode for six open reading frames (ORFs), namely the nucleocapsid (N), phosphoprotein (P), matrix (M), fusion (F), receptor binding protein (RBP) and large polymerase (L). The RBP is given alternate designations, including hemagglutinin (H), hemagglutinin-neuraminidase (HN) and attachment (G) protein for different taxa [1]. This family of viruses include several medically important genera such as *Morbillivirus, Respirovirus*, *Rubulavirus*, and *Henipavirus* [2]. Within these genera, there are several instances of zoonotic spillover, with notable examples being Hendra virus (HeV) and Nipah virus (NiV) that originated from *Pteropodidae* fruit bats [3].

*Jeilongvirus* was proposed as a new genus within the subfamily *Orthoparamyxovirinae* in 2018 following the discovery of its two initial members, Beilong virus (BeiV) and J-virus [4]. The *Jeilongvirus* genus is segregated from other members of the *Paramyxoviridae* family due to the presence of two additional proteins that encode a transmembrane (TM) and/or small hydrophobic (SH) protein, in addition to a larger than average G protein. However, this unique characteristic (TM and SH proteins) has also been found absent in two *Jeilongvirus* species, Mount Mabu Lophuromys virus 1 (MMLV-1) and Mount Mabu Lophuromys virus 2 (MMLV-2) from Rungwe brush-furred rats from Mozambique [5]. BeiV and J-virus were believed to have rodent origins due to their initial discovery in their host animals [6, 7]. Subsequent surveillance with pan-paramyxovirus PCR and metagenomics approaches uncovered a wider host range of *Jeilongvirus* [5, 8–11], with 15 established species [12] from bats, small mammals, and feline origin. Despite extensive reporting of *Jeilongvirus* diversity, their host specificity, mode of transmission and pathogenic effects remain understudied and largely unknown.

Small mammals such as rodents play an important role in maintaining virus genetic diversity but little is known about the viral diversity in Singapore. There are around 16 small mammal species in Singapore, and many of them inhabit peri-urban areas. Common and widely distributed species include *Mus castaneus, Rattus norvegicus*, and *R. tanezumi* [13]. The synanthropic lifestyle of small mammals, especially in a highly urbanized city like Singapore, creates opportunities for high contact interfaces with humans. This, in combination with small mammals’ competency as reservoirs for zoonotic diseases [14, 15], increases the risk for pathogen spillover and the spread of infectious diseases.

Although the zoonotic potential of *Jeilongvirus* is unknown, the diversity of infected species indicates potential past spillover events. Here, we investigated the prevalence of *Jeilongvirus* from small mammals in Singapore and elucidated the evolutionary relationships of three full length *Jeilongvirus* genomes retrieved in this study using a metagenomic approach. We also examined the ecology and epidemiology of this virus in Singapore by studying the relationships between host factors, such as species variety, life history traits and habitat types, and the likelihood of *Jeilongvirus* infection.

## Methods

### Sample collection

**Fig. 1:**
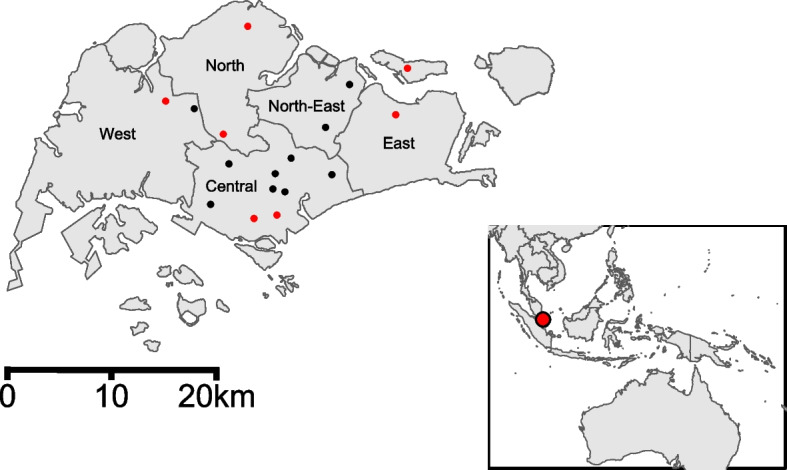
Map of Singapore showing the location of 17 sampling sites of captured small mammals. Red sites denotes location of *Jeilongvirus RT-*qPCR positive sampling sites, and black denotes *Jeilongvirus* negative sites. Inset map shows the location of Singapore within Southeast Asia

All animal work was conducted with approvals from the National University of Singapore Institutional Animal Care and Use Committee (IACUC B01/12), National Parks Board (Permit no NP/RP12-004) and the Agri- Food and Veterinary Authority of Singapore (Permit no AV16.01.004.0004). Small mammals (*n* = 100), including rodents and shrews, were captured using Sherman and Tomahawk traps from April 2012 to March 2016 from 17 locations across Singapore (Fig. 1). Trapped animals were identified to species using morphological features in addition to a COI barcoding PCR [16] when animals were unable to be morphologically identified. Individuals classified as pest species by Animal and Veterinary Science were euthanized using isoflurane. Both euthanized animals, as well as animals that died during handling were transported back to the laboratory for dissection in a biosafety level two cabinet. Lung, spleen and kidney tissues were collected and stored individually in viral transport media (VTM) or PBS and kept at -80 ºC until processed. Dissection instruments were disinfected with 1% virkon and 70% ethanol between harvesting each tissue and between individual animals.

### Sample processing and RNA extraction

For initial exploratory pathogen discovery, the lung, spleen, and kidney homogenates from 30 *Rattus tanezumi* individuals were pooled for metagenomic sequencing. For subsequent screening and next generation sequencing of individual small mammals samples, a 1–3 mm^3^ piece of individual lung, spleen and kidney from each animal was collected and separately weighed (2.0 to 32.2 mg). All tissues were homogenized in 500 µl of AVL buffer (Qiagen, Hilden, Germany) using silicon carbide shards and a Mini-Beadbeater-96 (Biospec Products, Bartlesville, USA) at 2000 rpm for 2.5 min. The homogenates were centrifuged at 12,000 rpm for 1 min, and 140 µl was used for RNA extraction with the Direct-zol RNA miniprep kit (Zymo Research Corporation, Irvine, USA) as per the manufacturer’s instructions.

### Metagenomics and virus full genome sequencing

Library preparation, sequencing and metagenomics analysis of the pooled *R. tanezumi* tissues were performed as previously described [17]. Briefly, the metagenomic library was prepared with the TruSeq RNA library preparation kit (Illumina, San Diego, USA) and sequenced on an Illumina MiSeq platform with 150 bp single-end reads. The subsequent FASTQ file was trimmed and taxonomically classified using DIAMOND v0.9 [18]. *Jeilongvirus* reads were extracted and mapped to a closely related genome sequence (MT085297) identified through BLASTn, with a full-length genome obtained and annotated for reference. This dataset is available at the NCBI Sequence Read Archive under BioProject accession number PRJNA990547. For whole genome sequencing of individual small mammal samples (*n* = 3), RNA was treated with the RiboZero Plus rRNA depletion kit (Illumina, San Diego, USA), prior to library preparation with the TruSeq RNA library preparation kit (Illumina, San Diego, USA) following the manufacturer’s instructions. The library was quality checked and sequenced on an Illumina MiSeq platform with 250 bp paired-end reads. FASTQ files were quality checked and trimmed with BBDuk2, before mapping to a reference genome created from the metagenomic dataset (PRJNA990547) to obtain the full-length genomes.

### Jeilongvirus detection and quantification

To screen for *Jeilongvirus* in the individual lung, spleen and kidney tissues of small mammals (*n* = 100), real-time qPCR (RT-qPCR) was performed using in-house designed primers, probe and positive control (179 bp minigene plasmid, pUCIDT, AMP) that targeted the *Jeilongvirus* L gene. The qPCR primers and probe were designed using Geneious Prime 2022.1.1. Unbiased metagenomic reads extracted from the *R. tanezumi* metagenomic dataset (PRJNA990547) were mapped to the previously obtained *Jeilongvirus* genome. Within the L gene region a 99% threshold was set for generating a consensus sequence, which did not include any ambiguities, that was used for oligo design.

The qPCR assay was optimized prior to screening with the AgPath-ID One Step RT-PCR kit (Applied Biosystems, Waltham, USA). A dilution range with triplicates of the positive control plasmid was used to generate a standard curve. The 25 µl reaction contained 0.5 µm of each forward (5’ - TTACAGGTCATGACGTCGGA − 3’) and reverse (5’ - TGGAATCTCGGGCTAGAGGG − 3’) primers, 0.25 μm probe (FAM - ACAATGAAAGGTTGACAAGCCA - BHQ1), 12.5 µl of 2 ´ buffer, 1 µl of 25 ´ reverse transcriptase enzyme mix and 5 µl of RNA template. The reaction profile was as follows: Reverse transcription at 50 °C for 15 min, followed by an initial denaturation step at 95 °C for 15 min and 45 cycles of 95 °C for 15 s and 54 °C for 30 s. Positive controls at an appropriate dilution were used in all qPCR runs. The optimized RT-qPCR assay generated a R^2^ of 0.995, an amplification efficiency (E) of 1.92, a limit of detection (LOD) of 144 copies per reaction and a limit of quantification (LOQ) of 425 copies per reaction. Viral copy numbers were measured in copies/mg based on the standard curve equation.

### Phylogenetic analysis

Phylogenetic analysis was conducted with *Jeilongvirus* genomes (*n* = 3) from this study and available *Paramyxovirus* sequences (*n* = 92) from GenBank. Multiple sequence alignments were performed using MAFFT v7 [19] in Geneious Prime 2022.1.1., and the L gene phylogenies were constructed with maximum likelihood method in RAxML v8 under the general time reversible (GTR + G) nucleotide substitution model. Branch support was assessed using 1000 bootstrap replicates. Phylogenetic analysis of the generated whole genomes was also constructed as mentioned above.

### Statistical analysis

We fit hierarchical Bayesian models with a Bernoulli distribution to reflect the temporal and spatial structure in our data and the binomial response variable of negative or positive for *Jeilongvirus*. To accommodate this hierarchical structure, we used multilevel regression models with a three-level structure, nesting individual positives within districts and years. The use of a varying effects model allows us to use partial pooling which provide better inferences on the estimates of prevalence, especially, in groups where sample size is low. Non-centered parameterization was utilized to navigate the posterior more efficiently. Priors were chosen to be weakly regularizing to control for both under- and overfitting of the model to the data. Convergence criteria, such as effective sample sizes and R-hat values, were used to check for appropriate model convergence throughout, and trace plots were inspected for signs of incomplete mixing when necessary. All models were fit with the ‘rethinking’ [20] package as a high-level interface to the Stan platform for statistical computing [21], model results and visualizations were conducted using the ‘tidybayes-rethinking’ [22] and ‘ggplot2’ [23]. All computing was conducted in ‘R’ [24]. To estimate the probability of RT-qPCR positivity among species, a three-level hierarchical Bernoulli distribution model was fit with district and year as the primary two levels and species, age, and sex as the tertiary level. To estimate the probability of RT-qPCR positivity among habitat types, a three-level hierarchical Bernoulli distribution model was fit with the interaction between district and year as the primary two levels and habitat as the tertiary level. For our numerically generated posterior samples, an estimated median probability with two-tailed 89% Bayesian credible intervals (BCI) for each parameter was reported (essentially, Bayesian confidence intervals).

## Results

A total of 100 small animals from eight rodent and shrew species were collected in Singapore from 2012 to 2016 (Fig. 1). The species were identified as *Mus castaneus* (*n* = 21), *Rattus exulans* (*n* = 2), *R. norvegicus* (*n* = 20), *R. sp.* (*n* = 1), *R. tanezumi* (*n* = 32), *R. tiomanicus* (*n* = 3), *Sundamys annandalei* (*n* = 15), and *Tupaia glis* (*n* = 6). Animals were captured from four habitat types and 17 locations across Singapore (Fig. 1). There were 53 males, 45 females and two animals of undetermined sex. Seventy-nine small mammals captured were identified as adults and the remaining were juveniles (Additional Table 1).

Metagenomic sequencing of the *R. tanezumi* pool yielded a total of 4,849,707 reads. Approximately 0.32% (*n* = 15,741) reads were classified as viral, of which 34.7% (*n* = 5,457) were classified as *Jeilongvirus*. Other viral reads detected in the *R. tanezumi* pool were bacteriophages and retroviruses. This dataset is available at the NCBI Sequence Read Archive under BioProject accession number PRJNA990547.

Twelve of 100 (12%) animals captured were RT-qPCR positive for *Jeilongvirus* (Table 1). Positive animals were from four species (*M. castaneus*, *R. exulans, R. tanezumi* and *R. tiomanicus)* that were trapped from May to August 2012, March 2013, September 2013, and December 2015, across seven sites (Fig. 1). *Jeilongvirus* was detected in spleen and kidney samples, but not in the lung tissues of all 100 animals. The kidney and spleen samples had an average viral copy number of 8.1 × 10^6^ and 3.4 × 10^4^ copies/mg respectively.

**Table 1:** Amplification cycle threshold (ct) values and viral copies per mg of tissues calculated from the RT-qPCR standard curve

Sample ID	Species	District	Collection Date	Spleen		Kidney	
				**Ct value**^a^	**Copies per mg spleen**	**Ct value**^a^	**Copies per mg kidney**
RTIO-2	*R. tiomanicus*	West	2012-06-08	29.6	40,986.8	25.7	179,632.8
RTIO-3	*R. tiomanicus*	West	2012-06-08	28.5	36,504.3	21.4	2,686,558.5
RE-1	*R. exulans*	Central	2012-08-28	-	-	23.4	1,501,978.1
RE-2	*R. exulans*	East	2015-12-11	-	-	21.0	4,253,155.6
RT-2	*R. tanezumi*	Central	2012-05-29	30.4	25,957.4	28.6	157,470.9
RT-5	*R. tanezumi*	Central	2012-05-26	-	-	21.5	4,297,980.4
RT-7	*R. tanezumi*	West	2012-06-05	-	-	18.8	16,334,042.9
RT-8	*R. tanezumi*	Central	2012-05-27	-	-	20.4	14,867,601.5
RT-16	*R. tanezumi*	North	2013-03-17	-	-	30.6	12,654.9
RT-19	*R. tanezumi*	North	2013-09-24	-	-	20.3	12,812,335.6
RT-21	*R. tanezumi*	North-East	2013-09-06	-	-	26.2	234,851.6
MU-21	*M. castaneus*	East	2015-12-06	-	-	30.4	4,842.4

- Ct value not detected; *CWC* Central Water Catchment, *CCK* Choa Chu Kang

^a^Limit of Quantification cut off at 32.08 (425.64 copies per reaction)

**Fig. 2:**
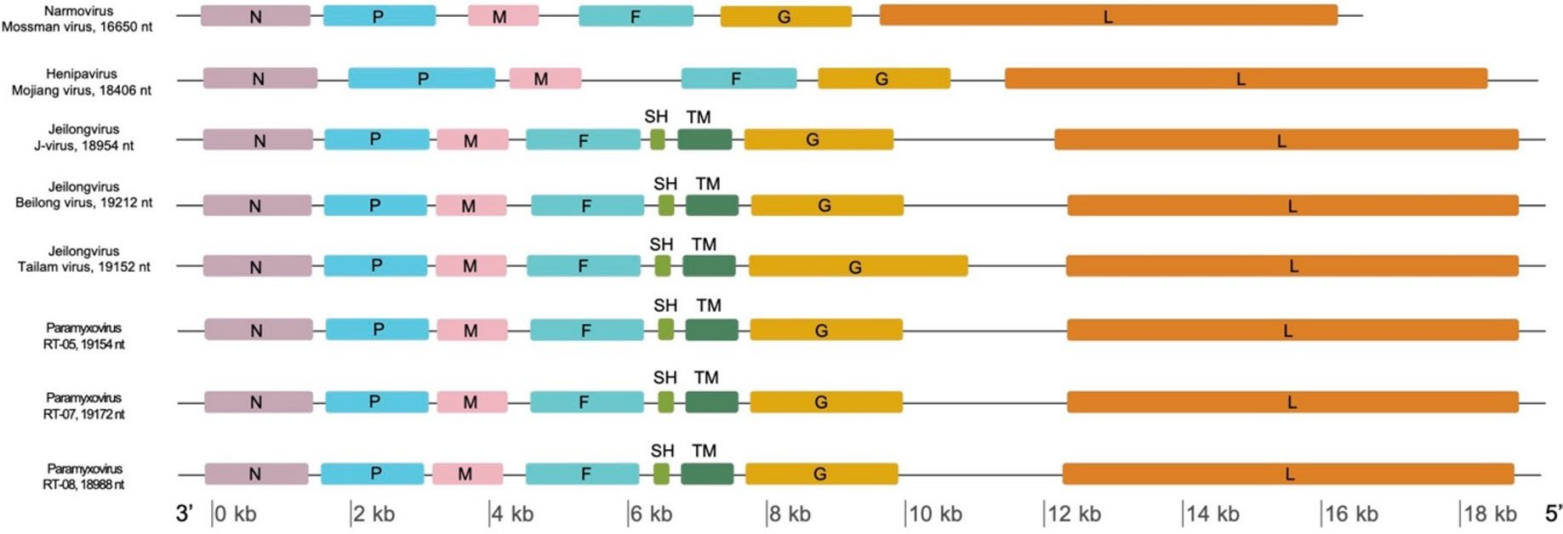
Genome organization of Singapore *Jeilongvirus* generated in this study and representative members of *Jeilongvirus* (AY900001, DQ100461, JN689227), *Henipavirus* (KF278639) and *Narmovirus* (AY286409). The Singapore *Jeilongvirus* genomes have eight open reading frames, which encode nucleocapsid (N), phosphoprotein (P), matrix protein (M), fusion protein (F), small hydrophobic protein (SH), transmembrane protein (TM), glycoprotein (G), and RNA polymerase (L). The figure is drawn to scale and the scale bar is shown at the bottom

Unbiased metagenomic sequencing was conducted on the 12 *Jeilongvirus* positive kidneys, with complete *Jeilongvirus* genomes recovered from three *R. tanezumi* samples (RT-05, RT-07 and RT-08). The total number of reads post-trimming and reads mapped to the reference genome are shown in Additional Table 2. The *Jeilongvirus* genomes from this study displayed a nucleotide similarity ranging from 97.1–100%, and an amino acid similarity of 97.7–100%. The annotated genomes have eight open reading frames (ORF) in the order: 5’- N/PVC/M/F/SH/TM/G/L -3’ (Fig. 2), consistent with the typical *Jeilongvirus* genome organization. The sequences generated in this study are available at NCBI Genbank (accession numbers OR233792 to OR233794) and as BioProject PRJNA990547.

**Fig. 3:**
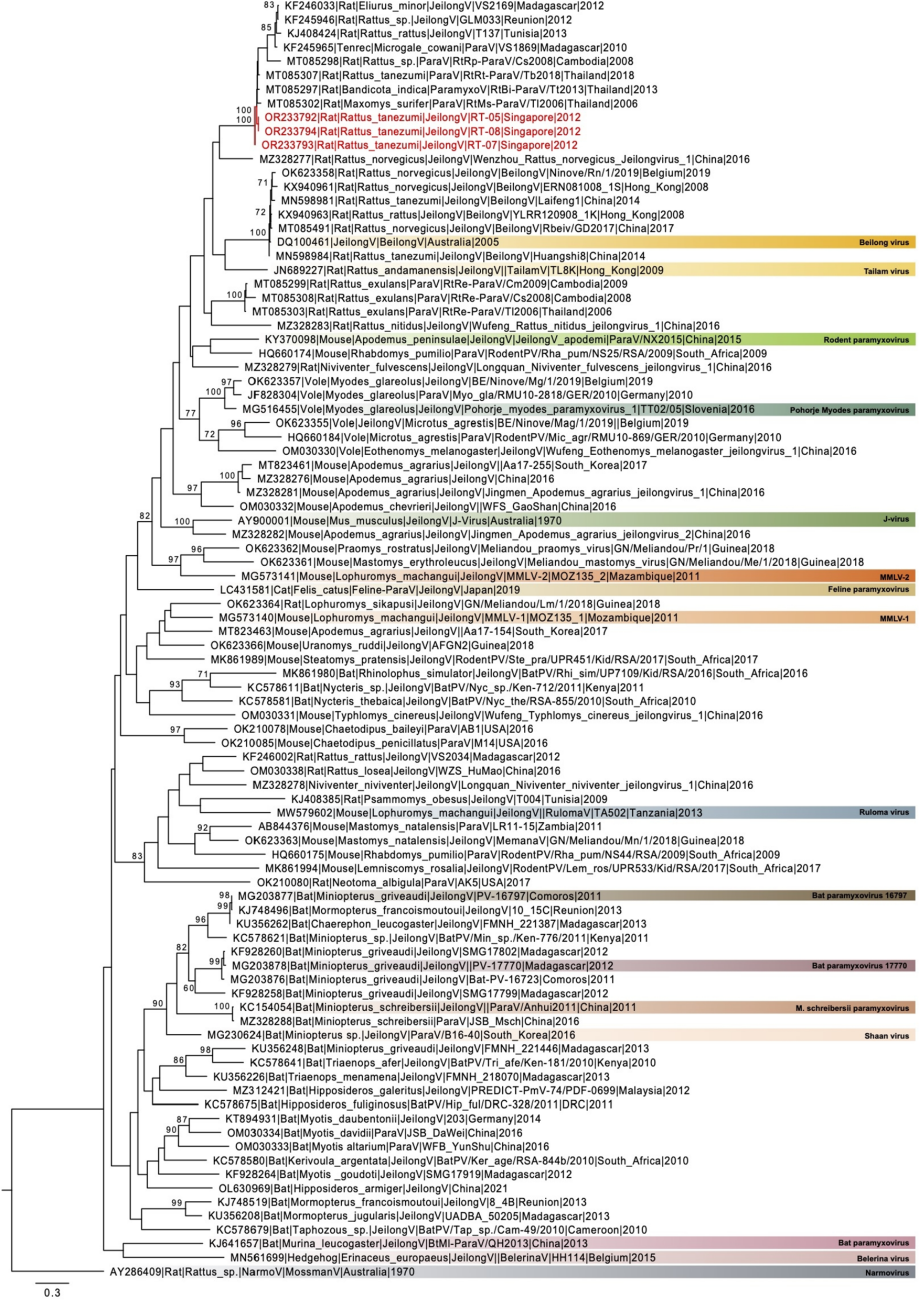
Maximum likelihood phylogeny of *Jeilongvirus* L gene sequences generated using the GTR + GAMMA model in RAxML. Coloured boxes indicate exemplar *Jeilongvirus* species. Red fonts indicate novel sequences generated from *R. tanezumi* in this study. Bootstrap values greater than 70.0% are indicated at branch nodes. The scale bar denotes nucleotide substitutions per site

Maximum likelihood phylogeny of the L gene reveals that the three *Jeilongvirus* species collected from *R. tanezumi* in Singapore clustered in a monophyletic clade with other unclassified paramyxoviruses identified in small mammals from Southeast Asia, Madagascar, Reunion, and Tunisia (Fig. 3), that is a sister clade to the *Wenzhou rattus norvegicus jeilongvirus 1*. Phylogenetic analysis of the full genomes (Additional Fig. 1) reflects similar phylogenetic relationships as the L gene phylogeny. The genomes of all three *Jeilongvirus* species identified from *R. tanezumi* also shared low genetic similarity with other representative virus species within the same genogroup, ranging from 55.0 to 70.1% nucleotide and 51.0–74.3% amino acid identities, respectively (Table 2).

**Table 2:** Genetic similarities between *Jeilongvirus* genomes from Singapore (*n* = 3) and related *Jeilongvirus* genomes

Sample ID	RT-05	RT-07	RT-08	RtBi-ParaV/Tt2013	TailamV	Wenzhou Rn jeilongV 1	BeilongV	J-virus
	Nucleotide Similarity (%)							
**RT-05**		97.09	99.97	95.82	70.50	70.14	68.05	55.80
**RT-07**	97.76		97.09	95.67	70.43	70.01	67.98	55.83
**RT-08**	99.97	97.73		95.81	70.45	70.11	67.95	55.70
**RtBi-ParaV/Tt2013**	96.63	96.64	96.63		70.49	70.08	67.83	55.77
**TailamV**	75.27	72.58	72.59	72.31		68.92	67.94	55.98
**Wenzhou Rn jeilongV 1**	73.69	73.58	73.82	73.50	71.77		68.00	55.23
**BeilongV**	71.75	71.71	71.68	71.44	71.88	71.17		55.32
**J-virus**	51.58	51.39	51.60	51.45	51.00	51.17	51.30	
	**Amino Acid Similarity (%)**							

**Fig. 4:**
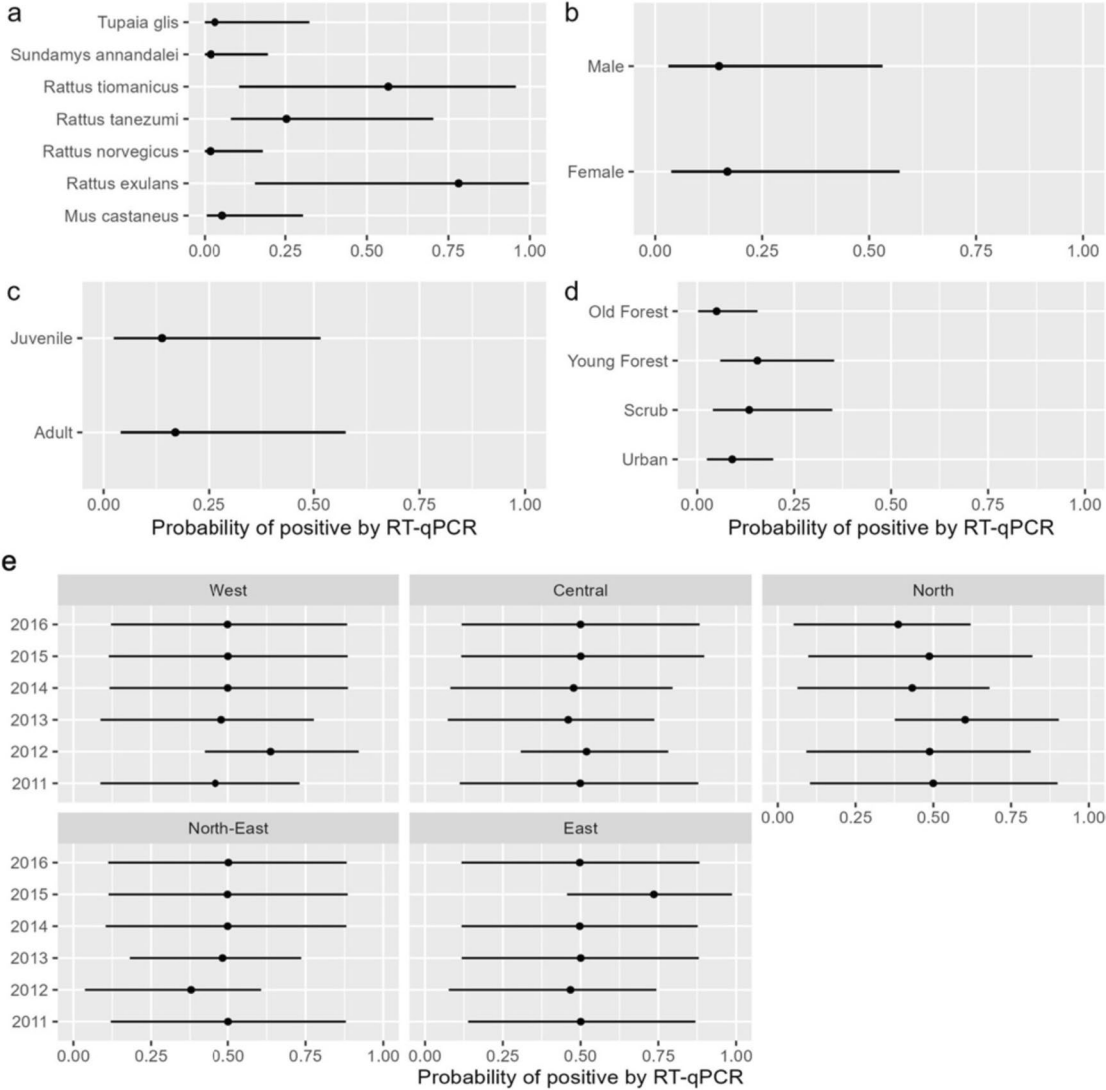
Results from Bayesian hierarchical models showing probability of RT-qPCR positive with 89.0% credible intervals for: (**a**) species, (**b**) sex, (**c**) age class (**d**) habitat, and (**e**) interaction term of year and district

Amongst the *Rattus* species in Singapore, *R. exulans* and *R. tiomanicus* showed higher probability of positivity for *Jeilongvirus*, although there was overlap of credible intervals (Fig. 4a). There were no differences in the likelihood of RT-qPCR positives across sexes and age classes (Fig. 4b and c). We also compared the likelihood of RT-qPCR positives among the four habitat types. Our data suggests that young forest and scrub habitats showed higher probability of *Jeilongvirus* positivity than urban and old forest habitats (Fig. 4d). There were no spatial or temporal patterns in the probability of positives (Fig. 4e), with overlapping credible intervals for the interaction term of district and year.

## Discussion

Several novel murine *Jeilongviruses* have been reported and sequenced in the last few decades, including J-virus, Beilong virus (BeiV) and Tailam virus (TaiV) [6, 7, 11, 25]. Continued discovery and characterization of viruses within *Jeilongvirus* have been augmented by global and national zoonotic surveillance programs, along with the increasing use of metagenomic sequencing. In this study, we detected *Jeilongvirus* in four small mammal species in Singapore, *M. castaneus, R. exulans, R. tanezumi*, and *R. tiomanicus*, suggesting a wide rodent host range. This is consistent with previous studies where *Jeilongvirus* detection occurred across a variety of small mammal species in countries such as Thailand, China, and Madagascar [26–28].

Phylogenetic analysis shows that the *Jeilongvirus* species divide into two major clusters, with the bats and felines falling in one cluster and small mammals in the other. The Singapore *Jeilongvirus* falls within the small mammal subclade, grouping with unclassified *Paramyxoviruses* from small mammals sampled in islands of the Indian Ocean and in Africa. Previous studies indicate a geographical and host correlation of J-virus and BeiV in mainland China, specifying that well-defined lineages circulated widely within their taxonomical host groups [26, 29, 30].

The results from the hierarchical model analysis showed no differences among species, life history traits, or habitat types. The first and second hierarchies in the model consisted of district and year which correspond respectively to spatial and temporal patterns in *Jeilongvirus* positivity, that were consistent in Singapore. The estimated posterior distribution from a Bayesian analysis is structured by the sample size of the data. For *M. castaneus*, where there was one positive individual, we can still estimate the probability of positivity, based on partial pooling, however the large variance is a result of the small sample size. We realize that it may be difficult to generalize this finding to the entire extent of *M. castaneus* distribution or among the species that we captured as part of this study. A larger sample size of *M. castaneus* may have given us more precise insight into these relationships. However, our initial findings can act as prior knowledge for future investigations into *M. castaneus* as a reservoir for *Jeilongvirus*.

There were no differences of positivity among small mammal life history traits such as sex and age, or among habitat types, although young forest and scrub habitats exhibited slightly higher positivity estimates than other habitats. Bayesian models perform well with unequal sample sizes since sample size is a component of the posterior distribution and is reflected in the variance components of the habitat positivity estimates. Larger sample sizes may have reduced the size of the credible intervals. By using a hierarchical Bayesian approach, we maximized the information in the data to arrive at the results presented. Our model results illustrate the generalist nature of this virus and can serve as a foundation for further study on the dynamics of *Jeilongvirus*.

At present, the pathology and clinical representation of *Jeilongvirus* remain unclear. A member species, J-virus, was first isolated in mice with hemorrhagic lung lesions [6] and its pathogenicity was investigated in mice experiments [31, 32]. While newly established member species have been identified in a variety of organs, including the heart, kidney, and liver [5, 11, 26], *Jeilongvirus* was detected mainly in kidney and spleen and was absent in the lung in this study, supporting the previous observations that *Jeilongvirus* potentially results in a systemic infection [26].

## Conclusions

There is reported evidence of human seroconversion to J-virus [25], however the zoonotic potential of *Jeilongvirus* remains unknown. As a compact and highly urbanized island, Singapore holds many human and animal interfaces. Robust routine surveillance including increased number of animals sampled and increased spatio-temporal extent of sampling across both populations, combined with virus isolation and serological studies is important to further improve our understanding towards this understudied genus and help instigate the management of a potential spillover.

### Supplementary Information

**Additional file 1: Additional Figure 1. **Maximum likelihood phylogeny of full Jeilongvirus genomes generated using the GTR + GAMMA model in RaxML. Coloured taxa represent exemplar Jeilongvirus species. Red fonts indicate novel genomes generated from Rattus tanezumi in this study. Bootstrap values greater than 70% are indicated at branch nodes. The scale bar denotes nucleotide substitutions per site.

**Additional file 2: Additional Table 1. **Metadata for captured small mammal individuals used for this study.

**Additional file 3: Additional Table 2. **NGS reads obtained from full genome sequencing of RT5, 7 and 8.

## Data Availability

All metagenomic data and sequences generated from this study are available in NCBI Genbank in BioProject PRJNA990547 and accession numbers OR233792 to OR233794.

## Abbreviations

BeiV: Beilong virus
F: Fusion
G: Attachment
L: Large polymerase
LOD: Limit of detection
LOQ: Limit of quantification
M: Matrix
MMLV-1: Mount Mabu Lophuromys virus 1
MMLV-2: Mount Mabu Lophuromys virus 2
N: Nucleocapsid
NGS: Next generation sequencing
ORFs: Open Reading Frames
P: Phosphoprotein
PBS: Phosphate buffered saline
RT-qPCR: Real time quantitative polymerase chain reaction
rRNA: Ribosomal RNA
SH: Small hydrophobic
TaiV: Tailam virus
TM: Transmembrane
VTM: Viral transport medium

## References

[1] Rima B, Balkema-Buschmann A, Dundon WG, Duprex P, Easton A, Fouchier R (2019). ICTV Virus taxonomy profile: Paramyxoviridae. J Gen Virol..

[2] Samal SK, editor. The Biology of Paramyxoviruses. Norwich: Caister Academic Press; 2011.

[3] Anderson DE, Wang L-F. Zoonotic Paramyxoviruses. In: Richman DD, Whitley RJ, Hayden FG, editors. Clinical Virology. 4th ed. Washington, DC: ASM Press; 2016. p. 949–66.

[4] ICTV proposal. 2018.011 M.A.v1. 2018 . Available from: https://talk.ictvonline.org/ictv/proposals/2018.011M.A.v1.Paramyxoviridae.zip. [Cited 28 April 2023].

[5] Vanmechelen B, Bletsa M, Laenen L, Lopes AR, Vergote V, Beller L (2018). Discovery and genome characterization of three new jeilongviruses, a lineage of paramyxoviruses characterized by their unique membrane proteins. BMC Genom.

[6] Jack PJ, Boyle DB, Eaton BT, Wang LF (2005). The complete genome sequence of J virus reveals a unique genome structure in the family Paramyxoviridae. J Virol.

[7] Li Z, Yu M, Zhang H, Magoffin DE, Jack PJ, Hyatt A (2006). Beilong virus, a novel paramyxovirus with the largest genome of non-segmented negative-stranded RNA viruses. Virol J.

[8] Noh JY, Jeong DG, Yoon S-W, Kim JH, Choi YG, Kang S-Y (2018). Isolation and characterization of novel bat paramyxovirus B16-40 potentially belonging to the proposed genus Shaanvirus. Sci Rep.

[9] Vanmechelen B, Meurs S, Zisi Z, Goüy de Bellocq J, Bletsa M, Lemey P (2021). Genome sequence of Ruloma Virus, a Novel Paramyxovirus Clustering Basally to members of the Genus Jeilongvirus. Microbiol Resour Announc.

[10] Vanmechelen B, Vergote V, Merino M, Verbeken E, Maes P (2020). Common occurrence of Belerina virus, a novel paramyxovirus found in Belgian hedgehogs. Sci Rep.

[11] Woo Patrick CY, Lau Susanna KP, Wong Beatrice HL, Wong Annette YP, Poon Rosana WS, Yuen K-Y (2011). Complete genome sequence of a Novel Paramyxovirus, Tailam Virus, discovered in Sikkim rats. J Virol.

[12] International Committee on the Taxonomy of Viruses (ICTV). : https://ictv.global/report/chapter/paramyxoviridae/paramyxoviridae/jeilongvirus.

[13] NParks. List of mammal species present in Singapore. 2015 [Available from: https://www.nparks.gov.sg/biodiversity/wildlife-in-singapore/species-list/mammal.

[14] Meerburg BG, Singleton GR, Kijlstra A (2009). Rodent-borne Diseases and their risks for public health. Crit Rev Microbiol.

[15] Mendenhall IH, Ch’ng L, Neves ES, Borthwick SA, Smith GJD (2018). High diversity of medically important gastrointestinal rodent-borne helminths in Singapore. Zoonoses Public Health.

[16] Ivanova NV, Clare EL, Borisenko AV (2012). DNA barcoding in mammals. Methods and Protocols..

[17] Low DHW, Ch'ng L, Su YCF, Linster M, Zhang R, Yan Z (2023). Cencurut virus: a novel Orthonairovirus from Asian house shrews (Suncus murinus) in Singapore. One Health.

[18] Buchfink B, Xie C, Huson D (2015). Fast and sensitive protein alignment using DIAMOND. Nat Methods.

[19] Katoh K, Standley DM (2013). MAFFT multiple sequence alignment Software Version 7: improvements in performance and usability. Mol Biol Evol.

[20] McElreath R. Statistical Rethinking: A Bayesian Course with Examples in R and Stan. Chapman and Hall/CRC. 2016.

[21] Stan Development Team. RStan: the R interface to Stan. R package version 2.26.9; 2022. https://mc-stan.org/.

[22] Kay M (2020). Tidybayes: tidy data and geoms for bayesian models. R Package Version.

[23] Wickham H. ggplot2: Elegant Graphics for Data Analysis. 2nd ed. New York: Springer; 2016.

[24] R Core Team. R: A Language and Environment for Statistical Computing. R Foundation for statistical Computing, Vienna, Austria. 2018. http://www.R-project.org.

[25] Jun M, Karabatsos N, Johnson R (1977). A new mouse paramyxovirus (J virus). Aust J Exp Biol Med Sci.

[26] Chen J-J, Zhang X-A, Fan H, Jiang F-C, Jin M-Z, Dai K (2020). Distribution and characteristics of Beilong virus among wild rodents and shrews in China. Infect Genet Evol.

[27] Wilkinson DA, Mélade J, Dietrich M, Ramasindrazana B, Soarimalala V, Lagadec E (2014). Highly diverse morbillivirus-related paramyxoviruses in wild fauna of the southwestern Indian Ocean Islands: evidence of exchange between introduced and endemic small mammals. J Virol.

[28] Wu Z, Han Y, Liu B, Li H, Zhu G, Latinne A, et al. Decoding the RNA viromes of rodent lungs provides new visions into the origin and evolution pattern of rodent-borne diseases in Mainland Southeast Asia. Microbiome. 2021;9:18. 10.1186/s40168-020-00965-z. 10.1186/s40168-020-00965-z PMC7818139 33478588

[29] Woo PC, Wong AY, Wong BH, Lam CS, Fan RY, Lau SK (2016). Comparative genome and evolutionary analysis of naturally occurring Beilong virus in brown and black rats. Infect Genet Evol.

[30] Zhang Y, Zhang J, Wang Y, Tian F, Zhang X, Wang G (2022). Genetic diversity and expanded host range of J paramyxovirus detected in Wild Small mammals in China. Viruses.

[31] Abraham M, Arroyo-Diaz NM, Li Z, Zengel J, Sakamoto K, He B (2018). Role of small hydrophobic protein of J paramyxovirus in virulence. J Virol.

[32] Li Z, Xu J, Chen Z, Gao X, Wang L-F, Basler C (2013). The L gene of J paramyxovirus plays a critical role in viral pathogenesis. J Virol.

