# Supplementary material for: Evolution and ecology of Jeilongvirus among wild rodents and shrews in Singapore
Source: One Health Outlook. 2023 Dec 18;5:19. doi: 10.1186/s42522-023-00094-1 (PMC10726567; doi:10.1186/s42522-023-00094-1)
Supplement: Supplementary file 1 — Additional file 1: Additional Figure 1. Maximum likelihood phylogeny of full Jeilongvirus genomes generated using the GTR + GAMMA model in RaxML. Coloured taxa represent exemplar Jeilongvirus species. Red fonts indicate novel genomes generated from Rattus tanezumi in this study. Bootstrap values greater than 70% are indicated at branch nodes. The scale bar denotes nucleotide substitutions per site. [file 42522_2023_94_MOESM1_ESM.pdf]

Additional Figure 1

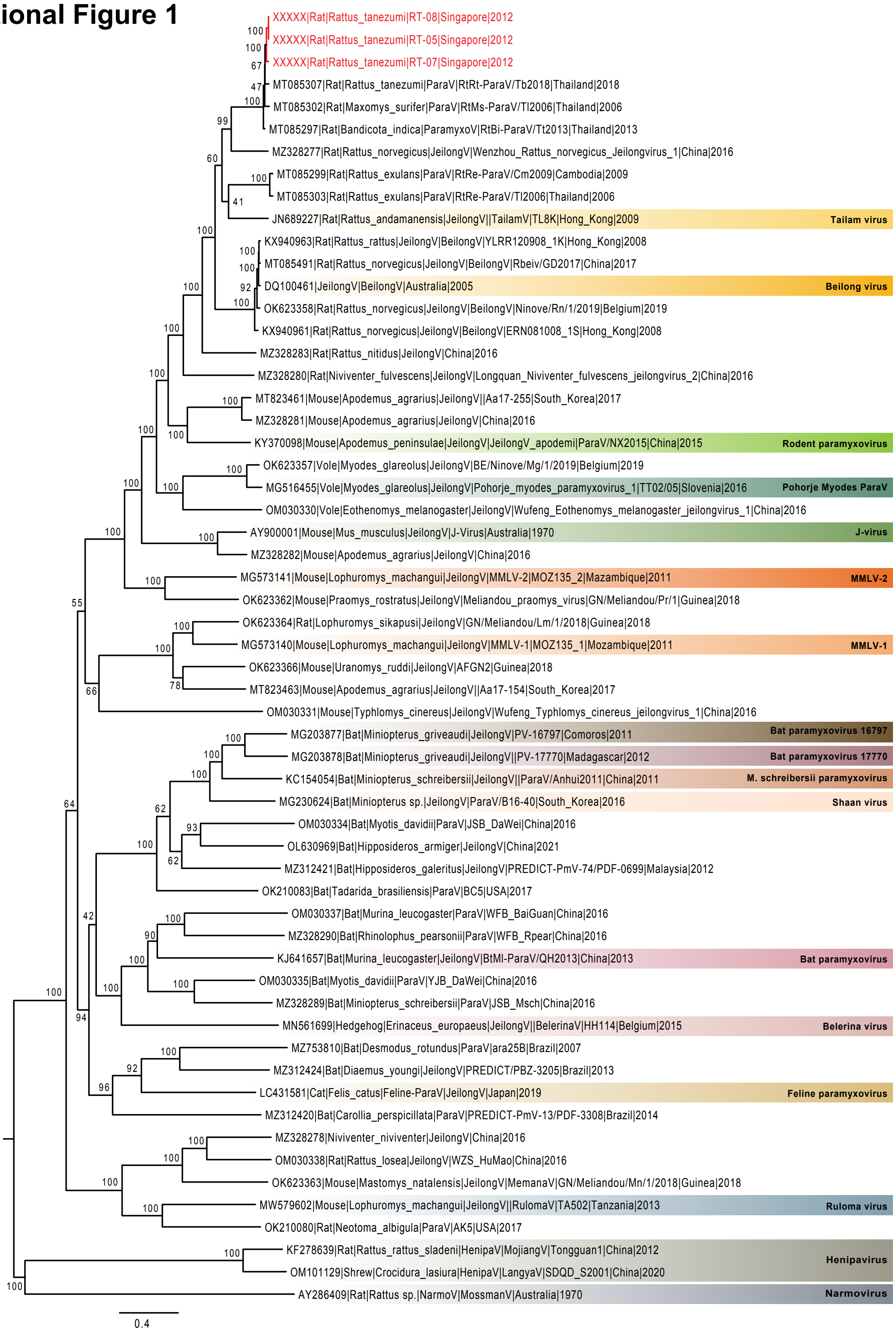

Maximum likelihood phylogeny of full Jeilongvirus genomes generated using the GTR + GAMMA model in RaxML. Coloured taxa represent exemplar Jeilongvirus species. Red fonts indicate novel genomes generated from Rattus tanezumi in this study. Bootstrap values greater than 70% are indicated at branch nodes. The scale bar denotes nucleotide substitutions per site.
