# Supplementary material for: Evolution and ecology of Jeilongvirus among wild rodents and shrews in Singapore
Source: One Health Outlook. 2023 Dec 18;5:19. doi: 10.1186/s42522-023-00094-1 (PMC10726567; doi:10.1186/s42522-023-00094-1)
Supplement: Supplementary file 2 — Additional file 2: Additional Table 1. Metadata for captured small mammal individuals used for this study. [file 42522_2023_94_MOESM2_ESM.pdf]

Additional Table 1. Metadata for captured small mammal individuals used for this study.

Sample ID	Species	Year of Collection	District	Location	Habitat Type	Age	Sex
RE-01	*Rattus exulans*	2012	Central	Bukit Merah	Young Forest	Adult	Male
RE-02	*Rattus exulans*	2015	East	Pasir Ris	Young Forest	Adult	Male
RTIO-01	*Rattus tiomanicus*	2012	West	Choa Chu Kang	Scrub	Juvenile	Female
RTIO-02	*Rattus tiomanicus*	2012	West	Choa Chu Kang	Scrub	Juvenile	Female
RTIO-03	*Rattus tiomanicus*	2012	West	Choa Chu Kang	Scrub	Adult	Male
TG-01	*Tupaia glis*	2011	West	Bukit Panjang	Scrub	Adult	Male
TG-02	*Tupaia glis*	2012	West	Bukit Panjang	Old Forest	Adult	Female
TG-03	*Tupaia glis*	2012	West	Bukit Panjang	Old Forest	Adult	Female
TG-04	*Tupaia glis*	2012	West	Choa Chu Kang	Scrub	Adult	Female
TG-05	*Tupaia glis*	2013	West	Bukit Panjang	Old Forest	Adult	Male
TG-06	*Tupaia glis*	2013	West	Bukit Panjang	Old Forest	Adult	Female
RN-01	*Rattus norvegicus*	2012	Central	Outram	Urban	Unknown	Unknown
RN-02	*Rattus norvegicus*	2012	East	Pasir Ris	Urban	Juvenile	Female
RN-03	*Rattus norvegicus*	2012	East	Pasir Ris	Urban	Adult	Male
RN-04	Rattus norvegicus	2012	Central	Toa Payoh	Urban	Adult	Female
RN-05	Rattus norvegicus	2012	Central	Toa Payoh	Urban	Adult	Female
RN-06	Rattus norvegicus	2012	Central	Outram	Urban	Adult	Male
RN-07	Rattus norvegicus	2012	East	Pasir Ris	Urban	Adult	Female
RN-08	Rattus norvegicus	2012	Central	Outram	Urban	Adult	Male
RN-09	Rattus norvegicus	2012	Central	Newton	Urban	Adult	Female
RN-10	Rattus norvegicus	2012	Central	Bukit Merah	Young Forest	Adult	Male
RN-11	Rattus norvegicus	2012	Central	Bukit Merah	Young Forest	Adult	Female
RN-12	Rattus norvegicus	2012	Unknown	Unknown	Unknown	Adult	Female
RN-13	Rattus norvegicus	2014	North	Central Water Catchment	Urban	Juvenile	Male
RN-14	Rattus norvegicus	2014	North	Central Water Catchment	Urban	Adult	Female
RN-15	Rattus norvegicus	2016	North	Sembawang	Young Forest	Adult	Female
RN-16	Rattus sp.	2016	North-East	Punggol	Urban	Adult	Male
RN-17	Rattus norvegicus	2016	North	Sembawang	Young Forest	Adult	Female
RN-18	Rattus norvegicus	2016	North	Sembawang	Young Forest	Adult	Female
RN-19	Rattus norvegicus	2016	North	Sembawang	Young Forest	Adult	Female
RN-20	Rattus norvegicus	2016	North	Sembawang	Young Forest	Adult	Female
RN-21	Rattus norvegicus	2016	North	Sembawang	Young Forest	Adult	Male
RA-01	Sundamys annandalei	2011	West	Bukit Panjang	Jungle Fall	Adult	Female

RA-02	Sundamys annandalei	2011	West	Bukit Panjang	Old Forest	Juvenile	Male
RA-03	Sundamys annandalei	2011	West	Bukit Panjang	Old Forest	Adult	Male
RA-04	Sundamys annandalei	2011	West	Bukit Panjang	Old Forest	Adult	Male
RA-05	Sundamys annandalei	2012	West	Bukit Panjang	Old Forest	Juvenile	Female
RA-06	Sundamys annandalei	2012	North	Central Water Catchment	Old Forest	Adult	Male
RA-07	Sundamys annandalei	2012	North	Central Water Catchment	Old Forest	Adult	Female
RA-08	Sundamys annandalei	2012	West	Bukit Panjang	Old Forest	Adult	Male
RA-09	Sundamys annandalei	2013	North	Central Water Catchment	Old Forest	Adult	Female
RA-10	Sundamys annandalei	2013	North	Central Water Catchment	Old Forest	Adult	Female
RA-11	Sundamys annandalei	2013	North	Central Water Catchment	Old Forest	Adult	Female
RA-12	Sundamys annandalei	2013	West	Bukit Panjang	Old Forest	Adult	Female
RA-13	Sundamys annandalei	2013	North	Central Water Catchment	Old Forest	Adult	Male
RA-14	Sundamys annandalei	2013	North	Central Water Catchment	Old Forest	Juvenile	Female
RA-15	Sundamys annandalei	2013	West	Bukit Panjang	Jungle Fall	Adult	Male
RT-01	Rattus tanezumi	2012	Central	Outram	Young Forest	Adult	Male
RT-02	Rattus tanezumi	2012	Central	Outram	Young Forest	Juvenile	Female
RT-03	Rattus tanezumi	2012	Central	Queenstown	Young Forest	Adult	Male
RT-04	Rattus tanezumi	2012	North-East	Punggol	Scrub	Adult	Male
RT-05	Rattus tanezumi	2012	Central	Outram	Young Forest	Adult	Male
RT-06	Rattus tanezumi	2012	West	Choa Chu Kang	Scrub	Adult	Male
RT-07	Rattus tanezumi	2012	West	Choa Chu Kang	Scrub	Adult	Female
RT-08	Rattus tanezumi	2012	Central	Outram	Young Forest	Adult	Female
RT-09	Rattus tanezumi	2012	Central	Bukit Merah	Young Forest	Juvenile	Male
RT-10	Rattus tanezumi	2012	Central	Outram	Young Forest	Adult	Female
RT-11	Rattus tanezumi	2012	Central	Outram	Urban	Unknown	Unknown
RT-12	Rattus tanezumi	2012	Central	Outram	Urban	Adult	Male
RT-13	Rattus tanezumi	2012	North-East	Punggol	Scrub	Adult	Male
RT-14	Rattus tanezumi	2012	Central	Outram	Young Forest	Juvenile	Male
RT-15	Rattus tanezumi	2013	North-East	Hougang	Young Forest	Juvenile	Male
RT-16	Rattus tanezumi	2013	North	Central Water Catchment	Urban	Adult	Male
RT-17	Rattus tanezumi	2013	North-East	Pulau Ubin	Young Forest	Adult	Male
RT-18	Rattus tanezumi	2013	North-East	Pulau Ubin	Urban	Adult	Female
RT-19	Rattus tanezumi	2013	North	Sembawang	Young Forest	Adult	Male

RT-20	Rattus tanezumi	2013	North-East	Pulau Ubin	Urban	Juvenile	Female
RT-21	Rattus tanezumi	2013	North-East	Pulau Ubin	Urban	Adult	Female
RT-22	Rattus tanezumi	2013	Central	Bukit Timah	Young Forest	Juvenile	Male
RT-23	Rattus tanezumi	2013	North-East	Pulau Ubin	Young Forest	Adult	Female
RT-24	Rattus tanezumi	2013	North-East	Pulau Ubin	Young Forest	Adult	Male
RT-25	Rattus tanezumi	2014	North	Central Water Catchment	Urban	Adult	Male
RT-26	Rattus tanezumi	2014	North	Central Water Catchment	Urban	Adult	Male
RT-27	Rattus tanezumi	2013	North-East	Pulau Ubin	Urban	Adult	Female
RT-28	Rattus tanezumi	2013	North	Central Water Catchment	Urban	Adult	Female
RT-29	Rattus tanezumi	2013	North-East	Pulau Ubin	Urban	Juvenile	Male
RT-30	Rattus tanezumi	2014	North	Central Water Catchment	Urban	Adult	Male
RT-31	Rattus tanezumi	2014	Central	Bukit Timah	Young Forest	Adult	Male
RT-32	Rattus tanezumi	2013	North-East	Pulau Ubin	Urban	Adult	Male
MU-01	Mus castaneus	2012	North-East	Punggol	Scrub	Adult	Male
MU-02	Mus castaneus	2012	North-East	Punggol	Scrub	Adult	Male
MU-03	Mus castaneus	2012	North-East	Punggol	Scrub	Juvenile	Female
MU-04	Mus castaneus	2012	North-East	Punggol	Scrub	Adult	Male
MU-05	Mus castaneus	2012	North-East	Punggol	Scrub	Adult	Male
MU-06	Mus castaneus	2012	Central	Novena	Urban	Adult	Female
MU-07	Mus castaneus	2012	Central	Geylang	Urban	Juvenile	Female
MU-08	Mus castaneus	2012	North-East	Punggol	Scrub	Adult	Male
MU-09	Mus castaneus	2012	North-East	Punggol	Scrub	Juvenile	Male
MU-10	Mus castaneus	2012	Central	Outram	Urban	Adult	Male
MU-11	Mus castaneus	2012	Central	Rochor	Urban	Adult	Male
MU-12	Mus castaneus	2012	Central	Queenstown	Young Forest	Adult	Female
MU-13	Mus castaneus	2012	Central	Queenstown	Young Forest	Juvenile	Female
MU-14	Mus castaneus	2012	Central	Queenstown	Young Forest	Juvenile	Male
MU-15	Mus castaneus	2013	North	Central Water Catchment	Urban	Adult	Female
MU-16	Mus castaneus	2013	Central	Bukit Timah	Young Forest	Adult	Female
MU-17	Mus castaneus	2013	North	Central Water Catchment	Urban	Adult	Male
MU-18	Mus castaneus	2014	North	Central Water Catchment	Urban	Adult	Male
MU-19	Mus castaneus	2014	North	Central Water Catchment	Urban	Adult	Female
MU-20	Mus castaneus	2015	North	Central Water Catchment	Urban	Adult	Male
MU-21	Mus castaneus	2015	East	Pasir Ris	Urban	Adult	Male
