# Supplementary material for: Evolution and ecology of Jeilongvirus among wild rodents and shrews in Singapore
Source: One Health Outlook. 2023 Dec 18;5:19. doi: 10.1186/s42522-023-00094-1 (PMC10726567; doi:10.1186/s42522-023-00094-1)
Supplement: Supplementary file 3 — Additional file 3: Additional Table 2. NGS reads obtained from full genome sequencing of RT5, 7 and 8. [file 42522_2023_94_MOESM3_ESM.pdf]

Additional Table 2. NGS reads obtained from full genome sequencing of RT5, 7 and 8.

Sample ID	Species	Tissue	# of raw reads	# of trimmed reads	# of mapped reads (to Reference Genome)	mapped reads /trimmed reads (%)	Genome Coverage (%)	Depth
RT-05	Rattus tanezumi	Kidney	783, 652	733, 254	11, 950	1.6297	99.97	234
RT-07	Rattus tanezumi	Kidney	1, 737, 634	1, 640, 416	101, 609	6.1941	99.99	1, 662
RT-08	Rattus tanezumi	Kidney	73, 938	61, 494	14, 412	23.4364	99.2	391
